# Biodegradable Bone Implants as a New Hope to Reduce Device-Associated Infections—A Systematic Review

**DOI:** 10.3390/bioengineering9080409

**Published:** 2022-08-22

**Authors:** José C. C. Paiva, Luís Oliveira, Maria Fátima Vaz, Sofia Costa-de-Oliveira

**Affiliations:** 1 Faculty of Medicine, University of Porto, 4200-319 Porto, Portugal; 2 DPS—Product Systems Development, INEGI—Institute of Science and Innovation in Mechanical and Industrial Engineering, 4200-465 Porto, Portugal; 3 IDMEC—Instituto Superior Técnico, Universidade de Lisboa, 1499-002 Lisboa, Portugal; 4 Departamento de Engenharia Mecânica, Instituto Superior Técnico, Universidade de Lisboa, 1499-002 Lisboa, Portugal; 5 Division of Microbiology, Department of Pathology, Faculty of Medicine, University of Porto, 4200-319 Porto, Portugal; 6 Center for Health Technology and Services Research—CINTESIS@RISE, Faculty of Medicine, University of Porto, 4200-319 Porto, Portugal

**Keywords:** bone temporary implant, antibacterial biomaterial, fixation devices, implant-associated infection, biodegradable implant, fractures

## Abstract

Bone fractures often require fixation devices that frequently need to be surgically removed. These temporary implants and procedures leave the patient more prone to developing medical device-associated infections, and osteomyelitis associated with trauma is a challenging complication for orthopedists. In recent years, biodegradable materials have gained great importance as temporary medical implant devices, avoiding removal surgery. The purpose of this systematic review was to revise the literature regarding the use of biodegradable bone implants in fracture healing and its impact on the reduction of implant-associated infections. The systematic review followed the PRISMA guidelines and was conducted by searching published studies regarding the in vivo use of biodegradable bone fixation implants and its antibacterial activity. From a total of 667 references, 23 studies were included based on inclusion and exclusion criteria. Biodegradable orthopedic implants of Mg-Cu, Mg-Zn, and Zn-Ag have shown antibacterial activity, especially in reducing infection burden by MRSA strains in vivo osteomyelitis models. Their ability to prevent and tackle implant-associated infections and to gradually degrade inside the body reduces the need for a second surgery for implant removal, with expectable gains regarding patients’ comfort. Further in vivo studies are mandatory to evaluate the efficiency of these antibacterial biodegradable materials.

## 1. Introduction

### 1.1. Infection

Osteomyelitis is a bone and marrow infection that can be developed due to a bloodstream infection or is usually secondary to a contiguous focus of infection in the context of trauma, reconstructive bone surgery or implant insertion [1,2]. Injuries such as open fractures can cause microorganisms to enter the body and infect the bone [2]. The incidence of osteomyelitis resulting from orthopedic trauma ranges from 5% to 10%, depending on the injury location and severity, type of fracture [3,4], the extent of collateral injuries, host’s physiological response and other risk factors, including male gender, smoking habits or diabetes mellitus [5,6,7,8,9,10]. The infection rate after internal fixation shifts between 1 and 2% on close fractures, rising to 30% on open fractures [11]. The two principal factors responsible for implant-associated infections are the immune ability at the interface between the implant and the surrounding tissues [12,13], which can be compromised after surgery [14], and biofilm formation, also associated with 80% of all chronic infections, according to the National Institutes of Health [15]. 

A biofilm is a conglomerate of bacteria held together and protected by a self-produced extracellular polymeric substance (EPS) matrix, composed of proteins, polysaccharides and extracellular DNA. The colonization of the bone or the implant occurs through bacteria adhesion and attachment. Then bacteria cells bind to each other and the extracellular matrix, maintaining biofilm integrity and mediating their communication through biochemical signals, increasing their resistance, namely, to available antibiotics, environmental stress and host immune response [16,17]. 

In general, *Staphylococcus aureus* and coagulase-negative staphylococci (CoNS) are responsible for up to two-thirds of all fracture-related infections, with *S. aureus* being the most prevalent single pathogen [18,19]. Other less common pathogens include members of the Enterobacterales family, *Pseudomonas aeruginosa*, *Streptococcus spp.* and anaerobes [18,19,20]. Polymicrobial infections can reach 30% of incidence and mainly occur in patients with open-fracture patients [20]. 

Multiple strains have developed high levels of antibiotic resistance [21,22], including MRSA, complicating the treatment of implant-associated infections and leading to poorer outcomes [23,24]. Although antibiotics are the first-line treatment for bacterial infections [25], sole treatment will frequently fail [26], as the inflammatory process leads to compression of vascular bone channels, causing ischemia and thus low penetration of antimicrobials into the inflamed bone tissue [27,28]. Therefore, tackling implant-associated infections usually requires a deeper and more invasive approach consisting of a secondary surgery involving irrigation, debridement and use of antibiotics as well, or even exchange or complete removal of the implant when necessary [29,30]. Besides being difficult to treat, implant-associated infections may represent a tremendous burden not just for patients receiving bone implants [31,32], considering the subsequent risk of a generalized infection [33], greater morbidity and psychological costs [34,35], but also from financial and societal points of view [25]. Bearing in mind how easily biofilms can thrive [33] and how difficult it is to treat implant-associated infections, the focus has been shifting towards the production of implants with intrinsic antibacterial properties to prevent implant-associated infections from arising [25].

### 1.2. Implants

All over the world, year after year, millions of people improve their lives through surgical procedures involving medical device implantation [29]. The implantable materials shall fulfill some requisites so that the surgery goals can be met, such as biocompatibility [36], not eliciting negative biological responses, and maintenance of an adequate function altogether [37,38]. Biomaterials used in medical implants are meant to improve their features [39,40]. The rising demand for implants and biomaterials across the globe, allied to incessant development in the field of Material Science and Engineering, has been responsible for great leaps forward in the medical implants field and their introduction into medical practice [29,36]. Not only in orthopedics but implants are also being used in other medical fields, whether in the form of catheters, pacemakers, cardiovascular stents, medication reservoirs, monitoring body functions, providing support and stimulating organs and tissues or even as tools for cosmetic or dental purposes [36,41,42,43]. The biomaterials used in implantable devices comprise metals, ceramics and polymers, based on metallic, ionic and covalent bonds, respectively [29], metals being the most preferred materials [44]. 

Metals have been famous for their mechanical strength for a long time. They have been used since the 1890s for orthopedic applications, incorporating high load-bearing implants. Stainless steel, titanium and cobalt-chromium alloys have dominated the market as orthopedic implant materials [44,45]. However, magnesium, a safely biodegradable in vivo and lightweight material, has recently been emerging as a good alternative due to its special properties, especially when combined with other elements such as calcium, zinc, manganese, strontium, tin and silver, which have been introduced to reduce corrosion and increase strength [36]. 

Polymers have been used in medical procedures as part of internal fixation devices [46], such as bone plates, screws and intramedullary pins [47]. They have overcome metals in some intrinsic properties, such as elasticity, flexibility, longevity and bio-inertness [48]. Among synthetic biodegradable polymers, the most widely employed have been polyglycolide (PGA), polylactide (PLA) and PCL [49,50], as they have good biocompatibility and are safely eliminated by metabolic pathways [51].

Ceramics are brittle materials, so they have limitations if used for load-bearing purposes. However, they can be useful while fixing or replacing hard connective tissues, including bone [38,52]. Calcium hydroxyapatite, besides being the bone inorganic phase’s main component [29], stands out among bioactive ceramics as a convenient coating for dental and orthopedic metal implants ensuring that their fixation in bone lasts longer [53].

Conventional non-absorbable or non-biodegradable implants also have some inherent disadvantages, including higher surgical risk and inconvenience and discomfort for patients taking into account the need for reoperations when implant removal is needed [46]. Implant failure may be due to mechanical factors, stress–strain imbalances, implant migration and wear debris, or biological reasons, such as foreign body reactions or bacterial infections [54,55,56]. According to the Center for Disease Control, the number of deaths caused by infections and antibiotic-resistant bacteria will outrun the fatalities from all kinds of cancers by 2050; hence, the development of medical devices comprising materials and properties capable of curbing bacteria functions is compelling [29].

Biodegradable or bioabsorbable implants have been revolutionizing orthopedic surgery, as long as they can be gradually replaced by new forming tissue, restoring the normal bone functions with no need to be removed in a second surgery [47]. Additionally, they can have integrated drugs or growth factors to promote healing and prevent infections [57,58,59]. However, since these devices are “programmed to disappear” inside the body and lose their biomechanical strength, any mismatch between the implant absorption and the healing process may have adverse results, including implant failure, fibrosis, or inflammatory reactions [60,61,62,63]. The implant absorption is not a guarantee of efficacy and once the absorption has started, it can be difficult to remove the device [64]. The biodegradable implants can only be employed in low load-bearing applications.

Herein, we systematically review the literature regarding the use of biodegradable bone implants in fracture healing and its impact on the reduction of implant-associated infections.

## 2. Methods

The systematic review was carried out in strict accordance with the Cochrane Handbook for Systematic Reviews of Interventions [65], and the Preferred Reporting Items for Systematic Reviews and Meta-Analyses (PRISMA) [66] checklist of the review was followed (Table S1 in Supplementary Materials).

### 2.1. Data Sources and Search Strategy

The electronic bibliographic databases of PubMed, Scopus and Web of Science were searched using a combination of MeSH terms and/or keywords in all fields of the research studies, regarding broad domains, such as osteomyelitis, bone fixation device, infection and biodegradable bone implant. For PubMed search, the following query was used: (Bone* OR “Bone and Bones”[Mesh]) AND (“Bone Infection*” OR Osteomyelitis OR “Implant-related Infection*” OR “Implant-associated Infection*” OR “Device-related Infection*” OR “Device-associated Infection*” OR “Bone Diseases, Infectious”[Mesh] OR biofilm*) AND (absorbable OR biodegradable OR bioabsorbable OR “Absorbable Implants”[Mesh] OR “Bioabsorbable Implant*” OR “Biodegradable Implant*” OR “Materials Testing”[Mesh]) AND (Device* OR Implant* OR “Bone-Implant Interface”[Mesh] OR “Fracture Fixation”[Mesh] OR “Osseointegration” OR “Prostheses and Implants”[Mesh] OR “Orthopedic Fixation Devices”[Mesh] OR “Bone Substitutes”[Mesh]). Regarding Scopus and Web of Science the used query was: (Bone* OR “Bone and Bones”) AND (“Bone Infection*” OR Osteomyelitis OR “Implant-related Infection*” OR “Implant-associated Infection*” OR “Device-related Infection*” OR “Device-associated Infection*” OR “Bone Diseases, Infectious” OR biofilm*) AND (absorbable OR biodegradable OR bioabsorbable OR “Absorbable Implants” OR “Bioabsorbable Implant*” OR “Biodegradable Implant*” OR “Materials Testing”) AND (Device* OR Implant* OR “Bone-Implant Interface” OR “Fracture Fixation” OR “Osseointegration” OR “Prostheses and Implants” OR “Orthopedic Fixation Devices” OR “Bone Substitutes”). Studies were selected and screened from January 2014 up to November 2021. The search included all publication types except reviews or systematic reviews and no language restrictions were applied.

### 2.2. Study Eligibility Criteria

Studies were included in this review if they met the following criteria: (1) in vivo or human or simultaneously both in vitro and in vivo studies, (2) full biodegradable implants/devices or scaffolds in bone fixation and its impact on infection prevention, (3) in vivo studies comparing biodegradable versus non-biodegradable implants regarding bone infection, (4) in vivo studies testing biodegradable implants and its antibacterial activity and (5) in vivo studies including microbiology analysis.

The exclusion criteria were: (1) studies conducted only in vitro, (2) studies not addressing bone infection and implant biodegradability; (3) biodegradable coatings on the surface of non-biodegradable implants; (4) studies that have no reference to implant applicability; (5) studies regarding only antibiotic delivery systems; (6) studies where no antibacterial testing or microbiology analysis was performed; (7) devices outside the scope of orthopedics; (8) reviews, systematic reviews, patents and grey literature.

### 2.3. Data Extraction and Search Results

**Figure 1:**
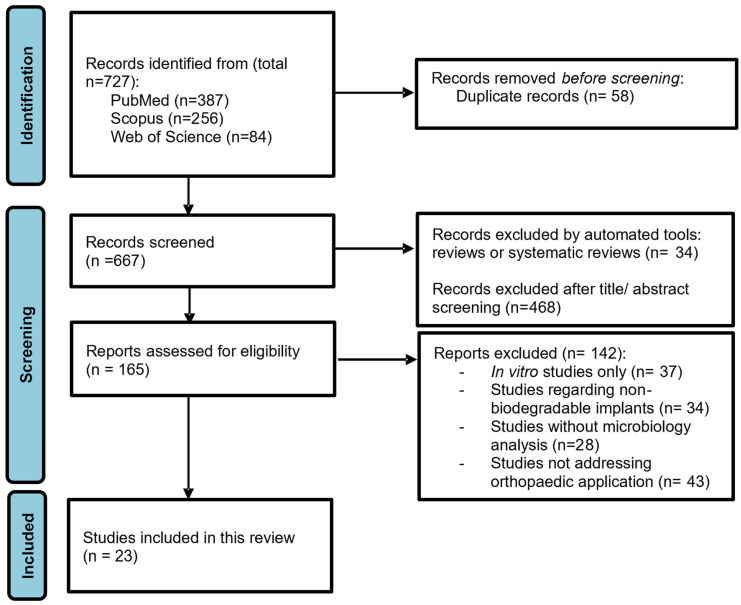
PRISMA flow chart representing the systematic identification of studies search via databases and registers.

**Figure 2:**
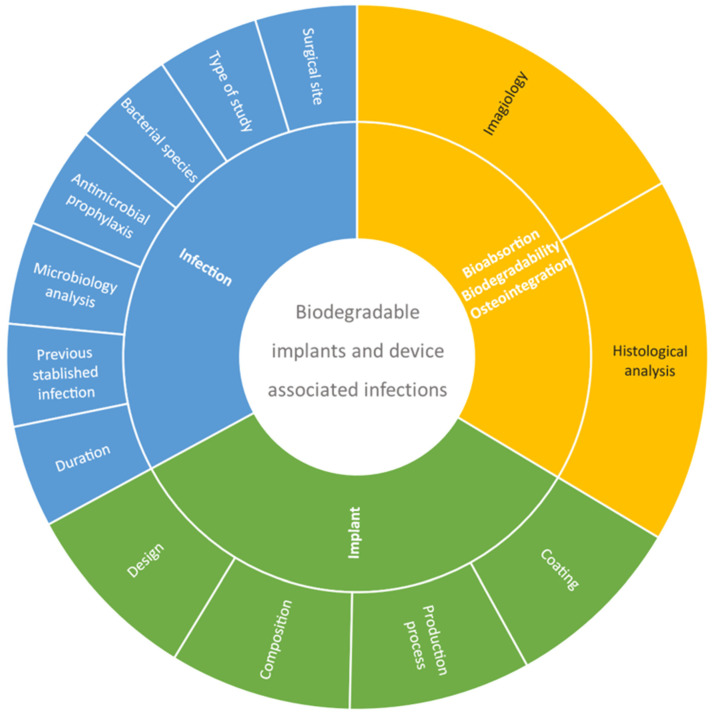
Systematic review outline strategy.

A total of 727 references were obtained in the three databases used: 387 from PubMed, 256 from Scopus and 84 from Web of Science. The extracted studies were uploaded to Rayyan software [67] for duplicate removal, quality assessment and further selection. A screening of the title, abstract and full text was performed and guided based on inclusion and exclusion criteria by two independent reviewers in a blinded standardized manner. Twenty-three studies were included in the systematic review (Figure 1). The outline of this systematic review is presented in Figure 2.

### 2.4. Assessment of Risk of Bias

To evaluate the risk of bias in the studies included in this review, the Office of Health Assessment and Translation Risk of Bias Rating Tool for Human and Animal studies were used. A 4-point scale was used to grade the potential source of bias as definitely low (++), probably low (+), probably high (−) or not reported (NR) and definitely high (−) (Table S2 in Supplementary Materials).

## 3. Results

### 3.1. Biomaterials

The implants used in the selected studies are present in Table 1 and Table 2. Different types and shapes, such as nails [68,69,70], screws [70,71,72], rods [73,74,75,76,77], wires [78], pellets [79], discs [80] and cylindrical or cubic scaffolds [81,82,83,84,85,86,87], were tested. A variety of materials were used, metals, polymers and calcium phosphates being among the most employed.

Mg was the most used metal, either alone in a pure state [72,74], or combined with other metals [69,73,74,76,77,78,88]. One study also reported a combination with a coating of UMAO-phytic acid [68]. Zn immediately followed Mg as the second most used metal [70,73,75,76,77,78]. Every study whose implants had a metallic composition incorporated at least one of these two components, and nearly half presented the combination of Mg and Zn in a Mg-Zn alloy [73,76,77,78]. The third most used metal was Cu, incorporated in Mg-Cu [69] and Zn-Cu [75] alloys. Others were employed less frequently, such as Ag [70], Ga and Sr [74], Al [73,76], Sn [77], Nd and Zr [88]. Most of these articles were comparative studies, using titanium implants as a control [69,70,72,73,74,75,77,78,88].

Apart from metallic devices, other studies used implants resulting from the mixture of different biodegradable polymers and calcium phosphates [71,79,81,83,84,85,86,87,89] or bone-like substitutes [82,85] as backbone materials, which could also incorporate macromolecules [81,89], nanoparticles [84] and silica [87]. Three implants followed a different pattern: two contained only a polymer within their core [80,85], and the other one was polymer-free [84]. The most used materials were poly-lactic acid-based polymers, PLGA, PLLA and PLDLA, as well as PU. Other polymers, such as PEG, PCL and Poly(3-hydroxybutyrate) (P3HB), were also employed. Among calcium phosphates, hydroxyapatite and nano-hydroxyapatite were the most prevalent.

### 3.2. Antimicrobial Agents

Some implants got their antibacterial properties conferred through several processes involving not just antibiotic impregnation, but also different combinations of antibiotics, metals, ions, polymers, salts, silica, hydroxyapatite or antimicrobial peptides, generally to produce device coatings or loadings (Table 1, Table 2 and Table 3). In other studies, implants contained materials within their core with intrinsic bactericidal activity, especially metallic elements (Table 2).

Vancomycin was the most widely used antibiotic, either impregnating the implant [85], combined with PLGA to cover [82] or incorporate [89] different scaffolds, or even combined with silica [83] for coating production purposes. Likewise, other antibiotics, such as imipenem/cilastatina (Tienam) [79] or a combination of gentamicin and clindamycin [81], were also used in scaffolds. Additional coatings were created by mixing levofloxacin with gold [90] and ciprofloxacin with PLLA and NaCl [91] (Table 2).

Apart from antibiotics, silver phosphate and silver nanoparticles were incorporated into scaffolds in two studies [84,86] and two additional coatings involving metals were produced through the combination of Cu with UMAO-phytic acid [68] and selenium with calcium phosphate [80]. Finally, a coating of PSI10, an antimicrobial peptide, combined with hydroxyapatite, was also investigated [76].

Taking into consideration the metallic implants, Mg and Zn were the most used ones, alloyed with each other and with other metals, boosting the antibacterial activity of the implants they integrated (Table 2).

**Table 1:** Implant type, production process and composition.

Ref.	Implant Type	Production Process	Control	Polymers	Calcium Phosfates			Bone			Macromolecules									Silica	Nanoparticles
				PLGA	PEG 400	PLLA	PLDLA	PU	PCL	P3HB	BCP	β-TCP	HA	n-HA	BHA	DPB	DBBP	CMC	PAA	MSN	RGO
[81]	Cylindrical Scaffold	Melt-blending, powder production and moulding		x	x							x						x			
[89]	Scaffold Granules	Emulsification-solvent evaporation; homogeneous method using an in-situ diffusion control system		x											x				x		
[82]	Cylindrical Scaffold	Bovine cancellous bone deproteinization; 3D Printing using Electrospinning		x												x					
[91]	Cylindrical Scaffold	Injection molding, hot melt dip coating				x															
[83]	Scaffold	3D printing using Electrospinning, aqueous precipitation of PLLA				x								x							
[71]	Screw	Mold injection process					x				x										
[86]	Scaffold	In situ foaming (method), Freeze-drying, mold\die production process						x						x							
[85]	Scaffold	3D printed Electrospun PU fibers						x					x				x				
[87]	Scaffold	3D printing of in-situ deposition of foams						x						x						x	
[80]	Disc	3D Printing, in situ precipitation							x												
[79]	Pellets	Bacterial biomass (bacterium Ralstonia eutropha B5786); Granulation, cold molding and water leaching	Bio-Oss^®^							x			x								
[84]	Scaffold	3D Precipitation in aqueous solution												x							x

Abbreviations: **PLGA**—Poly(DL-lactic acid-co-glycolic acid), **P3HB**—Poly(3-hydroxybutyrate), **BCP**—Bifasic-calcium phosfate, **β-TCP**—β-Tricalcium phosphate, **n-HA**—Nanohydroxyapatite, **BHA**—Bone-like hydroxyapatite/poly(amino acid), **Bio-Oss**—Bovine porous bone mineral xenograft, **DPB**—Deproteinized bovine cancellous bone, **DBBP**—Decellularized bovine bone particles, CMC—Carboxymethyl cellulose, **PAA**—Poly(amino acid), containing 6-aminocaproic acid, glycine, L-alanine, L-phenylalanine, L-proline and L-lysine, **MSN**—Mesoporous silica nanoparticles, **RGO**—Reduced graphene oxide.

**Table 2:** Implant type, production process and composition.

Ref.	Implant Type	Production Process	Control	Metals									
				Mg	Zn	Cu	Ag	Ga	Sr	Al	Sn	Nd	Zr
[68]	Nail	UMAO, Phytic acid conversion coating, electroless copper plating		x									
[72]	Screw	Additive Manufacturing, laser sintering, cold rolling	Ti	x									
[74]	Rod	Micro-alloying, powder metallurgy	cpTi	x				x	x				
[69]	Nail	Metallurgical casting	Ti	x		x							
[73]	Rod	Powder metallurgy	cpTi	x	x					x			
[76]	Rod	Solid-phase synthesis		x	x					x			
[77]	Rod	Powder Metalurgy, localized melting of Powders	Ti	x	x						x		
[88]	Cylinder	Additive manufacturing; selective laser melting	Ti	x	x							x	x
[78]	Wire	Powder metallurgy	Ti	x	x								
[75]	Rod	Extrusion of heat-treated materials	Ti		x	x							
[70]	Screw	Extrusion of heat-treated materials	Ti		x		x						

Abbreviations: **Mg**—Magnesium, **Zn**—Zinc, **Cu**—Copper, **Ag**—Silver, **Ga**—Gallium, **Sr**—Strontium, **Al**—Aluminum, **Sn**—Tin, **Nd**—Neodymium, **Zr**—Zirconium.

**Table 3:** Analyses of implants’ biodegradability, osteointegration and antibacterial properties: in vitro and in vivo studies.

Ref.	Implant Type (Scaffold, Screw, …)	Coating/Impregnated Antibiotics	Study Design/Type of Study	Surgical Site	Microorganisms	Previous Stablished Infection	Inoculation/Seeding of Microorganisms	Prophylatic Antimicrobial Admin.	Sacrifice Timepoint/Follow-Up Time after Surgery	Bioabsortion/Biodegradability	Osteointegration	Microbiology analysis In Vitro	Microbiology Analysis In Vivo	Outcome
[81]	Cylindrical Scaffold	None	In vivo	Femoral condyle	*S. aureus* (F2789)	No	At surgery	No	2 weeks	No	No	-	Presence of *S. aureus* (F2789)	The scaffold impregnated with 4% Gentamicin + 2.5% Clindamycin was effective at preventing S. aureus infection, whilst supporting a significant amount of new bone growth in a 13 week period.
		4% Gentamicin + 2.5% Clindamycin			*S. aureus* (F2789)		At surgery		2 weeks				No bacteria found	
		None			None		No		2 weeks					
		None			None		No		13 weeks	Yes	Yes			
		4% Gentamicin + 2.5% Clindamycin			*S. aureus* (F2789)		At surgery		13 weeks					
[89]	Scaffold granules	None	In vivo	Tibia metaphysis	*S. aureus*	Yes	Prior to surgery	No	4 weeks	-	No	-	-	The V-BHA/PAA scaffold promoted infection clearance and was gradually replaced by new forming bone during degradation. The scaffold and bone almost integrated with one another by the end of the experiment, and the bone defect underwent complete healing.
									8 weeks	-			-	
									12 weeks	-			-	
		Vancomycin-encapsulated in PLGA microspheres			*S. aureus*				4 weeks	-	Yes		-	
									8 weeks	-			-	
									12 weeks	Yes			Clearance of the infection	
		None			MRSA				4 weeks	-	No		-	
									8 weeks	-			-	
									12 weeks	-			-	
		Vancomycin-encapsulated in PLGA microspheres			MRSA				4 weeks	-	Yes		-	
									8 weeks	-			-	
									12 weeks	Yes			Clearance of the infection	
	PMMA Granules	Vancomycin			*S. aureus*				4, 8 & 12 weeks	No	No		-	
					MRSA				4, 8 & 12 weeks	No	No		-	
	None(Blank control group)	-			*S. aureus*				4, 8 & 12 weeks	-	-		-	
					MRSA				4, 8 & 12 weeks	-	-		-	
[71]	Screw	None	Case series	Tibia proximal tunnel	*P. aeruginosa*	Yes	No	Yes	2–4 years	No	Yes	-	No purulent discharge was observed	The soft-tissue reaction led to extrusion of still intact bioabsorbable screws.
[86]	Scaffold	None	In vivo	Tibia proximal metaphysis	*S. aureus* (ATCC 25923)	Yes	Prior to surgery (4 weeks)	No	3 weeks	-	Yes	-	Infection progression	n-HA/PU3 and n-HA/PU10 implants were equally efficient in reducing bone infection. There was no significant difference in bone remodeling between these 2 groups, although the degradation of n-HA/PU10 was faster.
									6 weeks	Partially				
									12 weeks	Yes				
		Ag (3%)							3 weeks	Very low	Yes	-	-	
									6 weeks	Partially			-	
									12 weeks	Yes			No significant bone infection symptoms	
		Ag (10%)							3 weeks	Partially	Yes	-	-	
									6 weeks	Yes			-	
									12 weeks	Yes			No significant bone infection symptoms	
	None(Blank control group)	-							3, 6 & 12 weeks	-	-	-	Infection progression	
[83]	Scaffold	Silica	In vivo	Femur diaphysis	MRSA (ATCC 43300)	Yes	Prior to surgery (1 week)	No	1 month	No	No	-	Severe infection	The nanocomposite scaffold with 15 wt% drug can undergo degradation and simultaneously control infection, even though 100% bacterial elimination was not observed. However, it showed higher antibacterial efficiency than the 5 wt% Vancomycin scaffolds.
									3 months	-	-		-	
		Silica + Vancomycin (SE-V5)							1 month	Mostly	-		Significant reduction in infection	
									3 months	Yes	Yes			
		Silica + Vancomycin (SA-V5)							1 month	Mostly	-			
									3 months	Yes	Yes			
		Silica + Vancomycin (SE-V15)							1 month	Mostly	-			
									3 months	Yes	Yes			
		Silica + Vancomycin (SA-V15)							1 month	Mostly	-			
									3 months	Yes	Yes			
[85]	Scaffold	None	In vivo	Radius middle shaft	*S. aureus* (UAMS-1)	Yes	At surgery	No	4 weeks	Yes	No	-	Presence of *S. aureus*	K20 Vancomycin-loaded scaffold prevented infection without compromising the bone regenerative properties of the scaffold itself. The scaffold utility would be compromised in an infected bone defect in the absence of antibiotic.
									8 weeks					
									12 weeks					
		Vancomycin							4 weeks	No	Yes		No signs of infection	
									8 weeks					
									12 weeks					
[87]	Cuboid Scaffold	Gold + Levofloxacin 1 mg	In vivo	Tibia medullary cavit	*S. aureus* (ATCC 25923)	Yes	Prior to surgery (4 weeks)	-	1 week	No	No	-	-	5 mg Lev@ MSNs/n-HA/PU began to degrade 12 weeks after implantation. Prior to 12 weeks, the integrity of the material structure provided mechanical support for bone repair and its degradation contributed to new bone formation. Infection signs were successfully curbed.
									3 weeks		Low		-	
									6 weeks		-		-	
									12 weeks	Partially	Yes		No signs of infection	
		Gold + Levofloxacin 5 mg							1 week	No	No		-	
									3 weeks		Low		-	
									6 weeks		-		-	
									12 weeks	Partially	Yes		No signs of infection	
	None(Blank control group)	-							1 week	-	No		Infection progression	
									3 weeks					
									6 weeks					
									12 weeks					
	PMMA cement	Levofloxacin 1 mg							1 week	No	No		-	
									3 weeks		-		-	
									6 weeks		-		-	
									12 weeks		Yes		No signs of infection	
		Levofloxacin 5 mg							1 week	No	No		-	
									3 weeks		-		-	
									6 weeks		-		-	
									12 weeks		Yes		No signs of infection	
[82]	Cylindrical Scaffold	PLGA + Vancomycin	In vivo	Radius diaphysis	MRSA (ATCC 25923)	Yes	At surgery	No	8 weeks	Yes	Yes	-	Lower bacterial load	ANDB scaffold possesd effective bactericidal activity against MRSA while promoting site-specific bone regeneration.
		PLGA						Vancomycin		Partially	Partially		Bacterial load in betwen	
		PLGA						No		No	No		Higher bacterial load	
	DPB	PLGA + Vancomycin	In vitro	-		-	-	-	Along 30 days	-	-	Antibacterial effect sustained for 28 days	-	
		PLGA										No antibacterial effect		
		None										No antibacterial effect		
[91]	Cylindrical Implantable matrice	PLLA (NPC) + Ciprofloxacin	In vivo	Femur	No	No	No	No	40 days	No	No	-	No signs of infection	PLLA based CPX-IMs with porous surface are compatible with surrounding bone and muscle tissues and can sustain adequate antibiotic concentrations within defected area, preventing infection. Nevertheless, CPX-IMs of larger pore size showed more successful osteointegration than the smaller pore sized.
		PLLA/NaCl40% (SPC) + Ciprofloxacin								Partilally	30–40% of surface area			
		PLLA/NaCl40% (LPC) + Ciprofloxacin								Mostly	60–70% of surface area.			
		Ciprofloxacin	In vitro	-	*S. aureus, Bacillus subtilis, Micrococcus luteus, E. coli, P. aeruginosa.*	-	Yes	-	Along 40 days	Partially	-	Strong activity within the first 4 days, with similar results and no diminution during the follow-up period from day 1 until 40.	-	
		PLLA (NPC) + Ciprofloxacin												
		PLLA/NaCl40% (SPC) + Ciprofloxacin												
		PLLA/NaCl40% (LPC) + Ciprofloxacin												
[68]	Nail	UMAO-phytic acid-Cu-0	In vivo	Mandible	No	No	No	No	2 weeks	In between	No	-	-	The introduction of Cu2+ in the copper plating coating effectively inhibited the growth and propagation of the bacteria, and the antibacterial rate was proportional to the Cu content. (However, the coating Cu-5 inhibited cell growth in vitro and was not evaluated in vivo.) Finally, UMAO-phytic acid-Cu 3min implants slow down the in vivo corrosion rate, promote antimicrobial activity and bone growth.
									4 weeks		Partially			
									6 weeks		Yes			
		UMAO-phytic acid-Cu-3							2 weeks	Lower	No			
									4 weeks		Yes			
									6 weeks		Yes			
		UMAO-phytic acid							2 weeks	Higher	No			
									4 weeks		Partially			
									6 weeks		Yes, but… (desorganized bone structure)			
		UMAO-phytic acid	In vitro	-	*S.aureus* (ATCC 6538)*E. coli* (ATCC 25922)	-	Yes	-	24 h	-	-	No antibacterial activity	-	
		UMAO-phytic acid-Cu-0										Low antimicrobial activity		
		UMAO-phytic acid-Cu-1										Antibacterial rate ~50%		
		UMAO-phytic acid-Cu-3										Antibacterial rate >90%		
		UMAO-phytic acid-Cu-5										Antibacterial rate >90%		
[80]	Disc	CaP	In vivo	Skull	-	No	No	-	8 weeks	Partially	Yes	-	-	The Se-CaP coating showed antimicrobial and bone-forming properties. The release of soluble HSe− ions from the Se nanoparticles strongly inhibited biofilm formation of *S. aureus*.
		Se-CaP								Partially	Higher			
		CaP	In vitro	-	*S. aureus* (ATCC 29213)	-	Yes	-	48 h		-	Extensive biofilm formation	-	
		Se-CaP									-	No bacteria growth		
[84]	None	-	In vivo	Radius middle shaft	MRSA	Yes	Prior to implantation (10 days)	No	4,8 & 12 weeks	-	No	-	-	The AHRG scaffolds effectively eliminated infection and inhibited biofilm formation. The scaffolds antibacterial capacity improved as the AgNP loading increased, becoming the strongest when 4% was reached.
	Scaffold	None							4,8 & 12 weeks		Low			
		4% AgNP							4,8 & 12 weeks		Yes			
		1%/2%/4%/8% AgNP	In vitro	-	MRSA	-	Yes	-	24 h		-	Excellent antibacterial performance for the 4% and 8% AHRG scaffolds.	-	
[76]	Rod	None	In vivo	Femoral condyle	-	No	No	Penicillin postop	4, 8 &12 weeks	-	No	-	-	HA coated AZ91 loaded with PSI effectively inhibited S. aureus growth while promoting the repair of bone function. The HA coating reduced the Mg allow corrosion; antimicrobial peptide incorporated into HA crystals had its activity retained.
		HA + PSI10							4, 8 & 12 weeks		Yes			
		HA							4, 8 & 12 weeks		To some extent			
		HA	In vitro	-	*S. aureus* (ATCC 25923)	-	Yes	-	Along 1 week	-	-	Lower antibacterial efficiency	-	
		PSI10										In between		
		HA + PSI10										Higher and retained antibacterial efficiency		
		HA	In vitro	-	-	-	-	-	Along 2 weeks	HA-coated Mg alloy showed lower degradation rate than bare Mg alloy	-	-	-	
		None												

### 3.3. Implant Antibacterial Properties, Biodegradability, Osteointegration: In Vitro and In Vivo Studies

#### 3.3.1. Microbiology Analysis

Bacterial species included in the in vitro and in vivo studies are displayed in Table 3. *S. aureus* was the most tested strain, including MRSA [82,83,84,89].

Regarding in vivo studies, to pre-establish infection in animal models, the majority of the animals underwent inoculation of bacteria either at the moment of the implantation surgery [81,82,85] or before the surgery [83,86,87,89], generally some few weeks previously. Dumlao et al. presented two human case studies reporting a PLDLA bioabsorbable screw extrusion and *Pseudomonas aeruginosa* tibial tunnel infection after 2 and 4 years of anterior cruciate ligament reconstruction [71]. *P. aeruginosa* that grow from patients’ tissue samples has shown to be susceptible to all antimicrobial drugs tested [71].

Concerning the in vivo studies complemented by in vitro relevant data on microbiology analysis, only one study reported previously established infection in animals before implantation (10 days) [84]. Moreover, one article evaluated the presence of infection in vivo, even though no animals had been intentionally infected [91]. The remaining studies did not address infection in vivo [68,76,80]. Finally, each one of the referred studies underwent further detailed microbiology analysis in vitro [68,76,80,84,91].

To assess the implants’ bacterial load and their effectiveness in preventing biofilm formation, the methods to evaluate the presence of bacteria along and at the end of the in vivo experiments were tissue culture [71,82,83], both tissue sample and blood culture [81], gross observation/appearance of bone defect [89,91], gross bone pathology [87], culture of bone and degrading scaffold [85], radiological evaluation through X-ray imaging and counting of white blood cells on venous blood analysis [86] and histopathological findings to exclude neutrophilic infiltrates and abscess formation [91]. Conversely, in vitro antibacterial analyses were obtained through the determination of inhibition diameter on agar plate culture [84,91], the growth of colonies on different films surfaces [68], crystal violet staining of biofilms formed 48 h after scaffold immersion on bacterial suspension [80], and through a liquid growth inhibition assay [76].

In in vivo experiments, the association of biodegradable implants with Ag nanoparticles [86] or with antibiotics such as gentamicin and clindamycin [81] and vancomycin [85] or even with antibiotics combined with other components, such as vancomycin with PLGA [82,89], vancomycin with silica [83], levofloxacin with gold [87] and ciprofloxacin with PLLA/NaCl [91], showed to significantly reduce [82,83,86] or eliminate the bacterial load at the surgical site [81,85,87,89]. The non-association of the implants to these complements led to lower or absent antibacterial efficacy [81,82,83,85,86].

In what concerns the in vitro experiments, the strategies that effectively contributed to increasing the device antibacterial rate were adding ciprofloxacin with or without PLLA and NaCl [91], UMAO-phytic acid-Cu-3 or 5 [68], Se-CaP [80], 4% and 8% Ag nanoparticles [84], or hydroxyapatite with PSI10 [76] to the implant coating or composition.

#### 3.3.2. Bioabsorption and Biodegradability

Several methods were implemented to evaluate the bioabsorption and biodegradability of the implants (Table 3), including gross pathology [71,83,87,89], bone histology, radiography [71,82,86,89], micro-CT [68,81,85,86,87] or sample weight after PBS immersion (in vitro) [76]. The follow-up period for in vivo studies ranged from 6 weeks [68] to 4 years [71], and the majority of the studies had a 12-week final time point [76,83,84,85,86,87,89]. The majority of the studies assumed a faster biodegradability rate as a desirable feature, while a few preferred an intermediate velocity [68,86] and others managed to find out ways to delay it [76,80,85].

The scaffolds containing a combination of poly-lactic acid derivatives with calcium phosphates or deproteinized bone particles showed different resorption rates in vivo, depending on the antibiotic incorporation within the implants, such as vancomycin [82,83,89], gentamicin and clindamycin [81], or none [71], as well as the integration of distinct components in the composition of the scaffold, or the follow-up period of each experiment, between 8 and 13 weeks after implantation [81,82,83,89], with one study reporting results from a 2–4-year-long period [81]. Regarding biodegradation outcomes, they ranged from complete [81] to partial [82,83,89] or no degradation [71] along the established experimental periods. After 13 weeks, the PLGA scaffold containing PEG 400, β-tricalcium phosphate and carboxymethyl cellulose (CMC) carrying 4% gentamicin and 2.5% clindamycin [81] had been totally degraded, being the only implant made of a poly-lactic acid derivative reaching complete degradation by the end of the experiment, among the analyzed studies.

Among the implants that were partially degraded at the end of the experimental period, it is relevant to highlight the vancomycin-eluting PLGA nanofiber-loaded DPB (ANDB) scaffold, revealing scarce material remnants after 8 weeks [82]. This study also showed that the resorption of the scaffold with no vancomycin incorporation was delayed, if the antibiotic had been exclusively given intravenously, or prevented when no antibiotic was administered. Moreover, across a 12-week-long period, the vancomycin-loaded bone-like HA/poly (amino acid) (V-BHA/PAA) scaffolds [89], with vancomycin-encapsulated PLGA microspheres, were all completely degraded, while the silica-coated Nanoha–gelatin/PLLA scaffolds with entrapped (SE-V) or absorbed (SA-V) vancomycin at 5%wt (SE-V5 or SA-V5) or 15%wt (SE-V15 or SA-V15) evidenced a more gradual degradation [83].

In the case study, in two patients with *Pseudomonas aeruginosa* tibial tunnel infection after 2 and 4 years of anterior cruciate ligament reconstruction, the 30% biphasic calcium phosphate and 70% PLDLA screws did not degrade in a 2-to-4-year period after surgery, with consequent transcutaneous extrusion of still intact bioabsorbable devices [71].

In a nutshell, among PU implants, the 10% Ag/n-HA/PU scaffold [86] and the K20 scaffold without vancomycin [85] had the fastest degradation rate, while the 1 or 5 mg Lev@ MSNs/n-HA/PU [87], the 0% Ag/n-HA/PU [86] and the K20/100 scaffolds had the slowest.

Finally, studies focusing on metallic implants used Mg or the Mg-Zn alloy within their core composition. Among the Mg nails, which were combined with UMAO-phytic acid, the implants with UMAO-phytic acid-Cu-3 showed lower degradation in 6 weeks, keeping the most complete morphology compared to Mg implants with UMAO-phytic acid-Cu-0 or UMAO-phytic acid (without Cu) [68], and a Mg-Zn alloy implant, namely, the AZ91 Mg alloy (Mg–9%Al–1%Zn) rod coated with HA, revealed a lower degradation rate than bare Mg alloy, in vitro, along 2 weeks [76].

#### 3.3.3. Osteointegration

Such as for bioabsorption and biodegradability, the methods used to assess implant osteointegration were gross pathology [83,87,89], bone histology [68,71,76,80,81,82,83,84,85,86,87,89], radiography [82,86,87,89], micro-CT [68,76,80,81,83,84,85,86,87] and even MRI (Table 2) [71,76,83].

Taking into account the scaffolds that contained poly-lactic acid derivatives combined with calcium phosphates or deproteinized bone, two studies had a 12-week follow-up period [83,89], while all the other studies [81,82,91] had different ending time points, ranging from 40 days [91] to 4 years [71]. Different osseointegration rates and abilities were found among the implants, depending on their impregnation with antibiotics, gentamicin and clindamycin [81], vancomycin [82,83,89] or ciprofloxacin [91] and the different elements included within their composition, as well as the presence of infection by different bacteria (Table 3).

With the shortest follow-up period of 40 days, the ciprofloxacin biodegradable implantable matrices (CPX-IMs) made of PLLA and coated with PLLA or PLLA/sodium chloride (40% NaCl) showed different osseointegration rates according to their coating’s pore size, no pores (NPC), small pore size (SPC) (150–250 um) or large pore size (LPC) (250–350 um). The NPC CPX-IM did not undergo osteointegration, and the SPC CPX-IM and LPC CPX-IM had 30–40% or 60–70% of their surface area covered by osteoid formation, respectively [91]. Thus, the larger the pore size, the more efficient the osseointegration was. In 8 weeks, the ANDB scaffold underwent osseointegration, with a newly formed trabecular structure and a bone callus largely repairing and bridging the bone defect. At this time point, the NDB scaffold, without incorporated vancomycin, although with intravenous prophylactic administration, had lower osteogenesis, while the NDB scaffold without vancomycin administration showed no osseointegration [82]. In 12 weeks, the V-BHA/PAA scaffold was almost completely replaced by bone, while the same scaffold without vancomycin did not osteointegrate [89]. In 13 weeks, the PLGA scaffolds containing PEG 400, β-tricalcium phosphate and carboxymethyl cellulose (CMC) carrying 4% gentamicin and 2.5% clindamycin evidenced osseointegration, presenting new bone ingrowth within the defect and areas of osteoid and calcified cartilage [81]. Finally, within 2–4 years, the 30% biphasic calcium phosphate and 70% PLDLA screws denoted closure of the previous tibia tunnel with newly grown bone [71].

Therefore, the implants that evidenced greater osseointegration efficacy were LPC CPX-IM in 40 days, the ANDB scaffold in 8 weeks, the 15%wt V-BHA/PAA scaffold and the 15%wt SE-V and SA-V scaffolds in 12 weeks, and the PLGA scaffolds containing PEG 400, β-tricalcium phosphate and carboxymethyl cellulose (CMC) carrying 4% gentamicin and 2.5% clindamycin in 13 weeks (Table 3).

Regarding implants incorporating a combination of polyurethane and calcium phosphates, the three studies that were conducted had a follow-up time of 12 weeks [85,86,87]. During that period, the n-HA/PU scaffolds showed different osseointegration properties. According to their Ag concentration (0%, 3% or 10%): the n-HA/PU scaffold without Ag (0%) showed a small amount of bone growing into the scaffold and bone destruction associated to the progression of the infection; the 3% Ag/n-HA/PU scaffold reported good bone healing, with large, heavy and mature new bone at the interface between the tibia and the materials, along with the voids and filling the bone marrow cavity; and the 10% Ag/n-HA/PU scaffold showed large and heavy new bone at the interface between the tibia and the scaffold, still having the presence of a dead cavity [86]. The K20/100 scaffold (loaded with vancomycin) preserved bone regenerative properties, fostering osseointegration with the bridging of the segmental bone defect, but no osseointegration was detected when no antibiotic was added to the scaffold [85]. Finally, the 1 and 5 mg Lev@ MSNs/n-HA/PU scaffolds showed gradual and slow new trabecular bone formation around the materials, with the newly formed trabecular bone surrounding and closely linked to the implants. However, the 5 mg Lev@ MSNs/n-HA/PU scaffold was associated with the highest new bone formation [87]. These findings suggest that the polyurethane and calcium phosphate scaffolds that provided a more efficient osteointegration within 12 weeks were the 3% Ag/n-HA/PU scaffold [86], the K20/100 scaffold [85] and the 5 mg Lev@ MSNs/n-HA/PU scaffold [87]. Two other implants were associated with efficient osseointegration: PCL discs coated with Se-CaP [80] and the 20% nHA@RGO scaffolds with 4% AgNP [84].

Finally, studies showcased implants made of metallic elements, with Mg [68] or Mg-Zn alloy [76] within their structure, and had distinct follow-up periods, namely, 4–6 weeks [68] or 4–12 weeks [76]. Within 6 weeks, the implants made of Mg UMAO-phytic acid (without Cu) or with Cu-0 or Cu-3 showed complete osseointegration, even though the scaffold without Cu led to a disorganized bone structure. Among the three, the scaffold with Cu-3 showed a faster osteointegration, within just 4 weeks [76]. Finally, the AZ91 Mg alloy rods coated with HA and PSI10 underwent osseointegration within 4 weeks, with good bone-promoting repair function; in 12 weeks, the lack of PSI10 led to just partial osseointegration [76].

### 3.4. Biodegradable vs. Non-Biodegradable: Comparative In Vitro and In Vivo Studies

#### 3.4.1. Microbiology Analysis

Several bacteria were tested across comparative studies (Table 4), in which the performance of biodegradable implants was compared to standard materials such as titanium and Bio-Oss^®^ (Bovine Porous bone mineral xenograft) [79]. MRSA strains were the most used bacteria to establish animal infection before the surgery [69,70,73,74,75,78,88]. Apart from *S. aureus*, the studies also evaluated *E. coli* O157:H7 (NTCC 12900) [77] and *Acinetobacter baumannii* (Ab307-0294) [73].

The microbiology methods to assess infection were implant culture, determination of colony-forming units (CFU) and biofilm formation in vitro and in vivo. One study compared P3HB pellets with or without Tienam, using Bio-Oss^®^ as a control. The P3HB pellets without Tienam reported the presence of *S. aureus* (44.1%) and associations of Gram-positive and Gram-negative anaerobic microorganisms and *E. coli* (55.9%) 30 days after implantation, even though a new evaluation at day 90 showed no bacteria [79]. P3HB pellets with Tienam were associated with no bacteria at these two microbial analysis timepoints, while Bio-OssBio-Oss^®^ was not able to eliminate the presence of *S. aureus* (48.2%) and associations of Gram-positive and Gram-negative anaerobic microorganisms and *E. coli* at both timepoints [79]. The remaining studies compared metallic implants with titanium implants (controls).

One retrospective cohort study compared patients with median malleolar fractures fixed with bioabsorbable Mg screws (23 patients) and with conventional titanium screws. In both, no evidence of deep infection in a period of 12 to 53 months after implantation was observed [72].

Four studies used Mg-Zn alloys to manufacture their implants [73,77,78,88], and some added to this alloy other metallic elements, including Al (AZ91) [73], Nd and Zr (JDBM BioMg alloy) [88], and 0.5Sn [77]. In two of these studies [73,78], the alloy and the Ti controls had similar effects on bacteria elimination, since they were not able to suppress infection, reporting the presence of *A. baumannii* (Ab307-0294) [73] or MRSA (ATCC 43300) [78] 7 days and 8 weeks, respectively, after implantation. Moreover, in one study, the control group, in which no bacteria were inoculated, reported no infection [78]. In another study, the JDBM BioMg alloy cylinder was associated with a small number of bacteria 4 weeks after implantation, compared to large amounts of bacteria present in the Ti control group at the same timepoint [88]. Moreover, in one study, three types of implants—a Mg-1Zn-0.5Sn rod, a Mg-1Zn rod and a Ti rod—were tested and compared regarding their antibacterial properties in vitro. The Mg-1Zn rod led to a low number of bacterial colonies. However, adding 0.5Sn to this Mg-1Zn alloy reduced even further the number of bacterial colonies [77], the Ti rod having the highest number of bacterial colonies among these three implants.

Other studies alloyed other different metallic elements with Mg. One study manufactured a Mg-0.1Ga-0.1Sr rod and compared it with simpler forms of the alloy, such as Mg-0.1Sr rod, Mg-0.1Ga rod and pure Mg [74]. The microbiology analysis was conducted 5 days after implantation. At this time point, the Mg-0.1Ga-0.1Sr rod was associated with few bacterial colonies, the Mg-0.1Sr and Mg-0.1Ga rods got some bacterial colonies, the pure Mg rod led to many bacterial colonies, and the Ti rod showed the highest number of bacterial colonies. Regarding the implant with Mg and 0.25Cu in an intramedullary nail, almost no bacteria have grown, 4 weeks after implantation, while Ti had multiple colonies, also confirmed through imagological findings [69].

Finally, two studies alloyed Zn with 2Cu [75] and 2Ag [70]. These alloys reported significantly lesser bacteria than Ti [75] and very few bacteria in the surrounding bone tissue and an absence of bacteria on the nail surface [70]. Two studies reported milder signs of infection; however, in the Ti implant controls, the nail surface and the bone tissues surrounding the nails contained multiple MRSA colonies within a biofilm matrix [70,75].

#### 3.4.2. Bioabsorption and Biodegradability

There was some variability in bioabsorption and biodegradability properties among the studied materials. Those properties were assessed in in vivo studies through histological analyses [70,75,79], visual examination [73], scanning electron microscopy (SEM) [74], X-ray [78] and MRI [69]. Some articles assessed their implant degradation ability in vitro, through implant incubation in simulated body fluid (SBF) [88], in Trypticase Soy Broth containing MRSA [78] or PBS [77]. The implant’s resorption follow-up time ranged from 5 days [74] to 53 months [72].

Among the comparative studies, most of the articles showcased metallic implants, whilst only one developed a polymer and hydroxyapatite composite-based implant [79]. This article developed P3HB pellets, loaded or not with HA. Both variants showed a slow degradation rate, even though the P3HB/HA composite implant had an even slower resorption rate [79]. Ninety days after surgery, the P3HB pellets were fully degraded, while the P3HB/HA pellets still had little remnants [79].

Regarding metallic implants, Mg was the most employed element. It was studied either alone or alloyed with other metals, usually aiming to delay its intrinsic degradation rate. One of the two studies that used bare Mg screws reported full degradation when analyzed across a 12–53-month period after surgery, preventing posterior implant removal procedures (0%) when compared to Ti implants (20%) [72]. Moreover, in another study, in 5 days post-op, pure Mg rods exhibited a high corrosion rate, which could be delayed when alloyed with 0.1Sr or a Mg-0.1Ga-0.1Sr [74].

The other studies in which Mg played a crucial role either alloyed it with Cu, in a Mg0.25Cu intramedullary nail partially degraded 4 weeks after implantation [69] or, in the majority of the cases, with Zn. The Mg-Zn alloy was employed in four studies, reporting a relatively slow absorption rate. Mg-Zn alloy Kirschner wires showed good corrosion resistance 2 weeks after implantation [78]. Moreover, 7 days after surgery, an AZ91 rod (with Al) showed a slower degradation than Mg, although evidencing corrosion products on its surface [73]. Moreover, in vitro analyses over 7 days revealed that a Mg-1Zn-0.5Sn rod had a slow degradation rate, since the SnO2 forming all over the implant surface helped prevent pitting corrosion [77], and Mg-Nd-Zn-Zr (JDBM BioMg alloy) cylinder [88] degradation decreased with the immersion time.

Regarding other Zn alloys, a Zn–2Ag screw used to fix a femoral condylar split-fracture showed many degradation products 3 months after surgery [70], and Zn-2Cu alloy cylindrical rods also evidenced degradation products 3–6 weeks after surgery [75].

#### 3.4.3. Osteointegration

Different material-based implants showed different osseointegration properties (Table 4). Every research in which those properties were assessed performed histological analyses [69,70,73,74,75,78,79,88]. Other complementary methods included X-ray [69,70,78,79,88], gross observation [69], MRI [69] and micro-CT [70,78]. Additionally, the observation period ranged from 4 weeks [69,88] to 120 days [79].

Most of the studies presented metallic implants, while only one came out with a composite-based implant, resulting from the combination of a polymer with HA [79]. In this study, both P3HB and P3HB/HA implants loaded with Tienam showed a more complete repair of the respective model bone defect than the commercial material Bio-Oss^®^, contributing to the support ability and functional recovery of the affected limbs [79]. Interestingly, the P3HB implants showed better biomechanical properties than P3HB/HA implants 60 days after surgery, whose developing cortical bone evidenced larger lacunae and was loosely structured; however, 90 days after surgery, the cortical bone had almost been completely formed [79].

When comparing osteointegration properties of Mg-Zn and Ti implants, Mg-Zn implants were better succeeded. One study referred to a greater bone-to-implant contact with the Mg-Zn Kirschner wires, in 8 weeks, since the corrosion layer formed on top of these implants leads to the development of a more favorable microenvironment for osseointegration and bone-implant integration than Ti [78]. Moreover, 4 weeks after implantation, Mg-Nd-Zn-Zr (JDBM BioMg alloy) implants revealed a higher volume of mineralized bone, higher trabecular number and lower trabecular spacing in the previously established defect than Ti implants [88].

Other Mg alloyed implants were also assessed showing promising results. Four weeks after implantation, Mg0.25Cu intramedullary nails led to bone defect repair through the regeneration of thin new trabecular bone, while Ti implants led to bone tissue destruction [69]. On the other hand, pure Mg, Mg-0.1Sr, Mg-0.1Ga, and Mg-0.1Ga-0.1Sr implants had a large number of newly formed fibrous tissue in their vicinity, 5 days after surgery, even though longer experiments are required to assess the Sr and Ga species role on bone tissue formation [74]. Additionally, Zn alloys also demonstrated interesting results towards osseointegration. Thus, 3–6 weeks after implantation, Zn-2Cu rods were associated with more mature new bone formation and higher bone mass, with less bone being resorbed, while Ti implants led to more immature new bone and more osteoclastic bone resorption [75]. Finally, both Zn–2Ag and Ti–6Al–4V screws showed great efficacy in fracture repair, within 3 months [70]. The Zn–2Ag screw inhibited osteoclast differentiation, promoting osseointegration with new bone tissue forming around the screws.

### 3.5. Risk of Bias Analysis

The twenty-three selected studies included 1 retrospective cohort study, 1 case series and 21 animal model studies, mainly in sheep (1), rat (9) and rabbit (11). None of these studies were excluded based on quality. The results of the risk of bias assessment included in the study are presented in the Supplementary Material section (Supplementary Material Table S2). Most of the studies showed low risk of bias practices and results. One study did not report whether caregivers and researchers were blinded [87]. Seven studies did not report randomization of the administration dose or exposure level [70,73,80,85,86,87,88]. One study did not report if allocations to study groups were adequately concealed [80].

## 4. Discussion

The repair of bone fractures usually requires internal fixation of bone fragments, with numerous sorts of devices, such as wires, pins, screws, plates, intramedullary nails or rods being commonly used to stabilize the fracture and restore early mobility, limb function or weight-bearing ability [92]. Nowadays, nondegradable metallic implants made of stainless steel or titanium alloys are usually the first-line choice for bone internal stabilization. However, the wear associated with long-term exposure to these materials can lead to some health complications, such as foreign body reaction or inflammation induced by the release of certain ions or particles [93], eventually resulting in a second surgery for implant removal, with a higher risk of infection and healthcare-associated costs [94]. Additionally, implant-associated infections can also elicit revision surgeries and debridement. To overcome these drawbacks, and thanks to innovation in the biomedical field, various biodegradable devices have been developed, some of them assuming intrinsic antibacterial properties, providing physical support for tissues, fostering tissue repair, regeneration or facilitating local drug delivery [95], including antibiotics. Biodegradable orthopedic implants are expected to gradually degrade at a pace compatible with the bone healing process while being slowly removed by the body [94].

A systematic review of the most recently published evidence over the last 5 years was conducted to evaluate whether biodegradable orthopedic implants could reduce the prevalence of implant-associated infections, while undergoing osteointegration, contributing to increasing the strength and biomechanical support in previously established bone defects and avoiding the need for second revision surgeries. To be included in this systematic review, the studies had to evaluate at least one of the following features in vivo: antibacterial activity, biodegradability or osteointegration, a requisite found across 23 studies.

The bacterial adhesion and biofilm formation around the implant’s surface is a leading cause of implant failure [96]. Since human pathogens’ resistance to antibiotics has been continuously increasing [86], the development of alternative ways to outrun the establishment of infection is required. Thus, the development of coatings with antibacterial activity conferred through some components other than antibiotics comes out as an attractive solution. Those components can be antimicrobial peptides, inorganic antibacterial metal elements or antibacterial polymers [96]. Coatings can be applied through some techniques, including electrodeposition [97], electrophoretic deposition [98], dip-coating [99], thermal spraying [100], chemical conversion or a biomimetic approach [101,102].

Besides infection prevention, coatings can also delay implant degradation and corrosion rate [68] and improve the osteoinductive and osteoconductive properties of the implant’s surface, strengthening the adhesion between the implant and the peri-implant newly formed bone [103]. Herein, we highlight some of the coatings explored in the reviewed studies we presume to be great alternatives to the use of antibiotics. Jiaqi et al., 2019, developed a coating of ultrasonic micro-arc oxidation phytic acid copper (UMAO-phytic acid-Cu) upon Mg implants. This coating was found to reduce bacterial propagation while decreasing the implant corrosion rate and promoting bone growth, in vivo [68]. Researchers also concluded that the higher the content of Cu2 + present in the coating film per unit time, the stronger its antibacterial properties, but also the stronger its cytotoxicity, inhibiting cell proliferation and osteoblast differentiation [68]. Hence, the copper-loaded time of 3 min was preferred to 1 or 5 min. The mechanism underlying copper antibacterial activity has to do with the interaction between positive copper ions and the negative charges on the bacterial cell surface, causing the copper ions to detach from the coating surface towards the bacterial cell membrane, and then through its body, destroying it [68]. Moreover, Jinhuan et al., 2014, investigated the AZ91 magnesium alloy coated with hydroxyapatite and an antimicrobial peptide, named PSI 10 [76]. Antimicrobial peptides are produced by all living species and belong to innate immunity, taking part in the primary host response against microorganisms [104], inducing lower antimicrobial resistance than conventional antibiotics [76]. This coating was able to sustain its antimicrobial effect throughout the experiment (7 days), as antimicrobial peptides incorporated onto HA crystals were slowly released, while evidencing a bacterial inhibition rate of over 50% after 4 days, in vitro [76]. In vivo, it promoted greater osteoblastic activity than pure and HA-coated magnesium alloys [76]. Since antimicrobial peptides have cationic properties, they selectively interact with bacteria cells, which are more negatively charged than mammalian cells [105]. Hence, antimicrobial peptides are biocompatible with osteoblasts, while evidencing substantial antibacterial activity against both Gram-positive and negative bacteria [106].

Finally, Cedryck et al., 2020, developed a two-layered, “bifunctional” coating on a 3D-printed (PCL) scaffold: the top layer, made of CaP, aiming to stimulate osteoblast cell functions, and the bottom layer, containing Selenium (Se) nanoparticles, aiming to release this antimicrobial agent [80]. This coating was able to inhibit *S. aureus* biofilm formation through the release of HSe ions [80]. Considering that only 15% of the ions were released after 3 weeks, it is expected that this coating will have a prolonged and sustained antibacterial performance [80]. Furthermore, Se has shown good osteogenic properties, increasing the expression of osteogenesis genes such as alkaline phosphatase, osteocalcin or collagen-I, promoting osteoblast differentiation or metabolism; this may explain why the Se-CaP coating was able to support higher bone formation than the CaP-only coating [80]. Interestingly, compared to silver nanoparticles, Se has much lower cell toxicity [107].

Even though the antibacterial activity of these coatings had only been evaluated in vitro, their favorable results in this field allied to good cell biocompatibility make them promising candidates to replace the use of antibiotics for preventing implant-associated infections. Another way found to curb infection without antibiotics was through the incorporation of silver nanoparticles within the implants’ core. Dongli et al., 2018, developed a composite scaffold whose core constitution contained silver phosphate particles [86], similar to Weizong et al., 2020, who included silver nanoparticles in their scaffold, also flanked by reduced graphene oxide (RGO) and n-Ha [84]. Silver ions released from hydroxyapatite create a bacteriostatic environment around the implant [108]. Their antibacterial performance resides in the ability to induce the degradation of the bacterial cell membrane, the ribosomes denaturation and preclude the bacterial DNA replication [109]. This bactericidal effect is achieved at low concentrations, as 35 parts per million (ppm), with no cytotoxicity for mammalian cells [110]. Both studies reported favorable results, in vivo. Within 12 weeks, the implants studied by Dongli et al., 2018, led to no significant bone infection symptoms [86] and the implant of Weizong et al., 2020, effectively eliminated the infection and inhibited biofilm formation [84].

Across the reviewed comparative studies, several metallic alloys containing either Mg or Zn or both were implanted in the context of infection, and their performance was compared to pure titanium, the most used material for permanent implants contacting with bone [96]. Mg alloys express a high chemical and electrochemical activity, therefore being prone to degradation in physiological environments [74]. The corrosion of Mg and consequent release of Mg^2+^ ions generate a rapid increase in pH in the peri-implant surrounding tissues, inducing toxic effects on bacteria. High alkalinity inhibits bacteria adhesion ability by decreasing their surface hydrophobicity [111], and disrupts the proton electrochemical gradient in the intermembrane space of bacteria, reducing the synthesis of ATP by excessive consumption of protons [112]. However, the fast degradation rate of Mg does not usually match the bone healing process, so it is suggested to alloy Mg with other metals to solve this impairment, increasing corrosion resistance and biomechanical properties, for an adequate fracture internal fixation [88]. On the other hand, Zn ions hold osteogenic properties, stimulating osteoblasts by promoting cell replication, alkaline phosphatase activity, synthesis of collagen and osteoblast marker gene expression [113,114,115]. Apart from the magnesium alloy AZ91 (Mg–9%Al–1%Zn) [73], which showed no antibacterial activity in vivo, in 7 days, the remaining studies evidenced a satisfactory antibacterial efficacy. The magnesium alloy AZ91′s slow corrosion rate hindered the production of a significant alkaline shift in pH in vivo, suppressing the implant’s antibacterial properties [73]. However, other studies, which included the Mg-Zn alloy within their implant’s composition, came to different conclusions: an Mg-Zn alloy implant [78] evidenced a significantly lower bacterial burden than Ti implants, although it did not eliminate these bacteria within 8 weeks, while an Mg-Nd-Zn-Zr implant (JDBM BioMg alloy) [88] showed no signs of infection within 4 weeks. One of the reasons to explain the apparent success of the JDBM BioMg alloy implant in inhibiting infection has to do with the high release of Mg^2+^ ions during the early implantation stage. Mg^2+^ activates macrophages in the pro-inflammatory M1 phenotype, which release pro-inflammatory cytokines and take part in the immune response against microorganisms, rather than the anti-inflammatory M2 phenotype, enhancing the phagocytic capacity [88].

Other alloys of Mg–Mg-0.1Ga-0.1Sr [74] and Mg0.25Cu [69] revealed high efficacy in tackling bacteria. The Mg-0.1Ga-0.1Sr implants [74] showed a slower degradation rate than pure Mg implants, with higher antibacterial activity and suppressing bacterial existence within 5 days [74]. Given that the Mg-0.1Ga-0.1Sr implant induced a lower pH increase and detained higher bactericide activity than pure Mg implants, Zhihan et al., 2019, concluded that the key factor for antibacterial performance was the release of Sr^2+^ and Ga^3+^ ions, rather than Mg^2+^ ions or pH increase [74]. Sr^2+^ ions can also play a role in bone cell growth, although a longer implantation period would be required to assess the mechanisms behind Ga and Sr species on osteogenesis [74]. On the other hand, the Mg 0.25 Cu implant was responsible for the suppression of bone infection within 9 weeks [69]. Besides antibacterial properties, Copper integrates many metalloenzymes with important functions in the human body and stimulates osteogenesis and angiogenesis, crucial for repairing bone defects as a result of infection [116]. The Mg0.25 Cu alloy presented an excellent antibacterial activity, hindering bacterial adhesion and biofilm formation, as well as bacterial virulence and MRSA genetic drug resistance [69].

To overcome some Mg drawbacks, such as fast corrosion rate, low mechanical strength, accumulation of released protons or pH elevation, some authors turned to Zn-based materials as an appealing alternative for orthopedic implants, attracted by their great mechanical properties, degradation dynamics and osteogenic ability [75]. Among the Zn alloys developed across the reviewed studies, Zn2Ag [70] and Zn2Cu [75] were linked to great antibacterial activity and milder signs of infection, due to their ability to prevent bacterial adhesion and biofilm formation. Zn alone also exerts some inhibitory effects on bacteria [75]. While the Zn-2Ag alloy was reported to down-regulate bacterial genes involved in adhesion, colonization, the biofilm-thickening phase, virulence and drug resistance [70], the Zn-2Cu alloy also offered protection against MRSA inflammatory and toxic effects [75]. Moreover, the addition of Cu to the Zn-2Cu alloy helped increase its strength while maintaining Zn ductility [75]. This alloy was associated with improved osteogenesis and cytocompatibility, with higher mature new bone and bone mass around the implant interface and little osteoclastogenesis [75]. On the other hand, although the Zn-2Ag alloy did not directly elicit osteogenic differentiation, its good biodegradability and ability to restrain inflammatory response caused by degradation products were responsible for actively promoting peri-implant osseointegration, besides inhibiting osteoclastic activity [70]. This alloy also exhibited excellent mechanical properties [70]. The Zn-2Ag alloy evidenced some advantages over other materials. For instance, organic polymers such as PGA, PLA, and PLLA/PDLLA produce acidic environments because of their degradation products, and Mg-based implants elicit an alkaline environment in the implantation site surroundings, leading to abnormal inflammatory responses [117,118]. Additionally, the Zn–2Ag alloy developed by Xinhua et al., 2021, evidenced significant superiority over other Zn-based alloys due to its ability to suppress infection and osteoclastogenesis and enhance osteointegration, mainly in low-immunity patients with a considerable prevalence of bone-related infections [70].

Some of the reasons for choosing metallic implants instead of implants made of polymers or hydroxyapatite have to do with the confidence metals provide in terms of resistance, load-bearing capacity and bone function restoration after trauma. However, some non-metallic implants developed across the reviewed studies revealed a great ability to fix unstable bone, with good osteogenic properties. The core material of these implants usually does not have intrinsic antibacterial properties, unless some antibiotics, metallic particles with anti-bacterial properties or other compounds with the same goal are incorporated within the implant.

Regarding implant manufacture, production processes used in the studies under focus spread over a myriad of different manufacturing approaches. The metal implants were mostly prepared using powder metallurgy, where the alloying is easier to process and fine tune. Concerning thermoplastics, the materials are also, tentatively, processed in powder forms, and consolidated using either solvents or hot forming. Depending on the applications, there is dominance on the application/shapes that are commonly processed for thermoplastics—scaffolds and discs. The dominant implant types for metal-based processes are mostly slim cylindrical geometries such as nails, rods and cylinders.

An analysis of the scoped results showcases that 3D printing (not additive manufacturing) is used at least in one of the production stages, either at defining the mold shape, or at producing the final part itself. Concerning metal-based implants, 3D printing is not yet a significantly used method. The design stage is still not sufficiently exploited, and thus the added value is not driving the printing process. In the case where selective laser melting was used [88], the cylinder walls were functionalized to take advantage of the intrinsically complex design possibilities. In May et al.’s work, the metal-based screw is 3D printed and post-processed with cold-rolling to attribute further mechanical properties [72]. This combination tackles a multi-objective purpose, where the enhanced mechanical properties driven by the cold rolling are exacerbated by the geometries\shapes of the screw walls. There is currently no other process that allows such complex designs, with such ease of manufacturing.

Three-dimensional printing and additive manufacturing in general will allow for, in the future, as inception showcased in this analysis, a combination of process performance with material performance as well as the design possibilities. These combinations will allow for Venn-like analysis where different combinations of materials, together with specific processes, will unlock new designs and thus new application domains.

All the metallic and non-metallic implants can be precursors and reflect the incessant search for the perfect orthopedic implant for internal bone fixation or bone regeneration. This hypothetic implant shall fulfil some features, including (1) a biodegradation rate consistent with the pace of new bone formation and native tissue regeneration [86], (2) intrinsic antibacterial and anti-biofilm properties, (3) biocompatibility and biosafety, precluding a strong inflammatory response and releasing degradation products well tolerated by the host [119], (4) fostering osteointegration and osteogenic properties, including with incorporation of osteogenic factors or precursors [120], (5) osteoconductive properties, with interconnected pores with adequate size, to allow for cell migration throughout the structure, as well as transport of waste products (in the case of a scaffold) [120], (6) fixation to the surrounding tissues, allowing for stability and load transfer [121], (7) surface roughness on a nanometer scale to promote cell adherence [121], (8) mechanical properties similar to the bone, including strength and weight-bearing ability, and (9) filling the bone void, mimicking the true native tissue morphology to guide the regeneration accordingly (in the case of a scaffold) [120].

The present work faced some limitations. On the one hand, most of the reviewed studies did not state a clear distinction between the concepts of biodegradable and bioabsorbable implants, so these terms were considered to have the same meaning in our analyses. On the other hand, the majority of studies did not have a follow-up period long enough for the natural development of infection, so the microorganisms were artificially seeded into the surgical site and therefore the infection was pre-established before surgery. There was only one study [72] in which no bacteria were inoculated, with 48 patients being followed for a 12–53-month period of time, implanted with either Mg or Ti implants, even in this case, and despite the kind of implant, no signs of infection were detected during that period, although Ti implants underwent a higher removal rate due to pain or difficulty in shoe-wearing. Additionally, the different studies employed different methods to evaluate implant efficacy regarding biodegradability, osteointegration and antibacterial activity: some studies did not address all these variables and others evaluated some features only in vitro rather than in vivo. Moreover, some relevant aspects of the implantation success, such as the biosafety of implants and the impact of their degradation products on the organism as a whole or in specific organs, were not exhaustively addressed in this work. Finally, the existence of a timeframe in the inclusion criteria and the fact that no metanalyses were carried out also stand out as two additional limitations.

## 5. Conclusions

Implant-associated infections are among the leading causes of failure of orthopedic implants, usually leading to a second revision surgery with additional costs for healthcare systems and discomfort for patients. The present systematic review made an overview of the most recent literature about biodegradable orthopedic implants, with the ability to be gradually absorbed within the organism, provide mechanical and structural support for the unstable bone lesion and foster the elongation of new bone while hindering the development of bacterial biofilms. Among metallic implants, the Mg- and Zn-based alloys were the most attractive materials, usually combined with other metallic elements such as Ag or Cu, among others, with intrinsic antibacterial activity. Other biopolymers and hydroxyapatite-based implants were also assessed, usually associated with innovative coatings or other components within the implant core structure with antibacterial properties. These implants are expected to be a safe alternative to reduce the need for second surgical procedures since they proved to be efficient in preventing the development of infection and to be replaced by regenerative bone tissue throughout the healing process. Furthermore, the current application and landscape observed in this analysis can be defined as conservative, from a production process standpoint. Casting and traditional metallurgical processes are still dominant, as observed, and the authors expect that new technologies such as additive manufacturing can have a decisive and acute effect on the applications of biodegradable implants and medical devices. From the design process to the laser sintering or thermoplastic fusion processes, soon the process-related advantages will be showcased in literature.

A great number of studies were excluded from the present work for only taking their experiments in vitro, and most of the included studies carried out their experiments in animal models, rather than in humans. Thus, future in vivo works, especially those pertaining to humans, are required to transfer the acquired knowledge on biodegradable implants onto Orthopedics clinical practice.

## Figures and Tables

**Table 4:** Analyses of implants’ biodegradability, osteointegration and antibacterial properties across comparative studies.

Ref. #	Implant Type (Scaffold, Screw, …)	Study Design/Type of Study	Surgical Site	Microorg.	Previous Infection	Inoculation/ Seeding of Microorganisms	Prophylatic Antimicrobial Admin.	Sacrifice Timepoint/Follow-Up Time After Surgery	Infection		Bioabsortion/ Biodegradability			Osteointegration		Main Outcome
									Microbiol. Analysis	Histological Analysis	Imagiology (Radiographic, ect…)	Histological Analysis	Imagiology (Radiographic, ect…)	Histological Analysis	Imagiology (Radiographic, ect…)	
[79]	P3HB pellets	In vivo	Tibia metaphysis	*S. aureus*	Yes	Prior to surgery (1 month)	Chlorhexidine [##]	15 days	-	-	-	-	-	-	-	P3HB/HA and P3HB led to quicker suppression of infection and recovery of the support ability of the affected limb than Bio-Oss^®^. However, P3HB/HA composite implants showed inferior biomechanical properties than P3HB since cortical bone had large lacunae and was rather loosely structured. P3HB-based materials showed pronounced osteoplastic properties and slow degradation in vivo, enabling normal reparative osteogenesis.
								30 days	*S. aureus* (44.1%) and associations of Gram-positive and Gram-negative anaerobic microorganisms and *E. coli* (55.9%)	-	-	Partially	-	Partially	-	
								60 days	-	-		-	-	-	Yes	
								90 days	No bacteria	-	-	Yes (low rate)	-	Yes	Yes	
								120 days	-	-	-	-	-	-	Yes	
	P3HB/HA pellets + Tienam							15 days	-	-	-	-	-	-	-	
								30 days	No bacteria	-	-	Partially (lower rate)	-	Partially	-	
								60 days	-	-	-	-		-	Yes	
								90 days	No bacteria	-	-	Mostly	-	Yes	Yes	
								120 days	-	-	-	-	-	-	Yes	
	Bio-Oss^®^ (control)							15 days	-	-	-	-	-	-	-	
								30 days	*S. aureus* (48.2%) and associations of Gram-positive and Gram-negative anaerobic microorganisms and *E. coli*	-	-	-	-	-	-	
								60 days	-	-	-	-	-	-	-	
								90 days	*S. aureus* (48.2%) and associations of Gram-positive and Gram-negative anaerobic microorganisms and *E. coli*	-	-	-	-	No	No	
								120 days	-	-		-	-	-	No	
[73]	AZ91 rod	In vivo	Humeral head	*A. baumannii* (Ab307-0294)	Yes	Just before implantation	No	7 days	No antimicrobial effect	-	-	-	Partially *	-	-	AZ91 did not produce antimicrobial effects.
	c.p. Ti rod (control)								No antimicrobial effect	-	-	-	No *	-	-	
[74]	Mg-0.1Ga-0.1Sr rod	In vivo	Femur medullary cavity	*S. aureus* (ATCC 43300)	Yes	Just before implantation	No	5 days	Few bacterial colonies	-	-	-	Yes (lower)**	Yes	-	Mg alloys outperformed c.p. Ti in inhibiting *S. aureus* on the rods surface. Such antibacterial activity was improved through addition of micro-content of Ga and Sr (0.1 wt%).
	Pure Mg rod								Many bacterial colonies	-	-	-	Yes (higher)**	Yes	-	
	Mg-0.1Sr rod								Some bacterial colonies	-	-	-	Yes (lower)**	Yes	-	
	Mg-0.1Ga rod								Some bacterial colonies	-	-	-	Yes (higher)**	Yes	-	
	c.p. Ti rod								Highest number of bacterial colonies	-	-	-	No **	No	-	
	c.p. Ti rod (negative control)			-	No	-			No bacteria	-	-	-	No **	No	-	
	None (positive control)			*S. aureus* (ATCC 43300)	Yes	Just before implantation			-	-	-	-	-	-	-	
[72]	Mg screw	Retrospective cohort study	Tibia medial malleolus	-	No	-	-	Mean time of 24.6 ± 10.5 months (12–53 months)	No deep infection	-	-	-	Yes	-	-	Bioabsorbable Mg and titanium screws had similar therapeutic efficacy in MM fracture fixation. There was no implant removal with Mg screws.
	Ti screw								No deep infection	-	-	-	No	-	-	
[75]	Zn-2Cu cylindric rod	In vivo	Femur medullary cavity	MRSA	Yes	Soaked in implants	No	3 and 6 weeks	Significantly lesser bacteria were found	Few bacteria	Milder signs	-	-	Yes	-	The Zn-2Cu alloy exerted effective bacterial-killing capability and inhibited the inflammatory and toxic side-effects induced by MRSA bacteria in the rat femur.
	Ti cylindric rod								Large amounts of bacteria	Large amount of bacteria	Yes	-	-	No	-	
	None			-	No	-			-		No	-	-	-	-	
[88]	Mg-Nd-Zn-Zr (JDBM BioMg alloy) cylinder	In vivo	Distal femur	MRSA	Yes	-	-	4 weeks	Small number of bacteria	-	No	-	-	-	-	The JDBM BioMg alloy implant showed antibacterial properties against MRSA, decreasing biofilm formation.
	Ti cylinder								Large number of bacterial colonies	-	Yes	-	-	-	-	
[69]	Mg0.25Cu intramedullary nail	In vivo	Tibial metaphysis	MRSA	Yes	Prior to surgery (4 weeks)	No	4 weeks	Almost no bacteria	-	-	-	Partially	Yes	-	The Mg0.25Cu alloy demonstrated antibacterial properties and a therapeutic effect in chronic tibial osteomyelitis.
								9 weeks	-	-	-	-	-	-	-	
	Ti intramedullary nail							4 weeks	Multiple bacterial colonies	-	Yes	-	No	No	-	
								9 weeks	-	-	-	-	-	-	-	
[78]	Mg-Zn alloy Kirschner wires	In vivo	Distal femur	MRSA (ATCC 43300)	Yes	Just before implantation	No	2 weeks	Presence of MRSA	-	-	-	-	-	-	Better bone-implant integration was observed around the Mg-Zn alloy implants compared with Ti in the absence of MRSA. The corrosion product layer deposited on the surface of the Mg-Zn alloy implant retarded the corrosion of the implant, promoting osteointegration.
								4 weeks		-	-	-	-	-	-	
								6 weeks		-	-	-	-	-	-	
								8 weeks		-	-	-	-	Yes	Yes	
				-	No	-		2 weeks	No bacteria	-	-	-	-	-	-	
								4 weeks		-	-	-	-	-	-	
								6 weeks		-	-	-	-	-	-	
								8 weeks		-	-	-	-	Yes	Yes	
	Ti Kirschner wires			MRSA (ATCC 43300)	Yes	Just before implantation		2 weeks	Presence of MRSA	-	-	-	-	-	-	
								4 weeks		-	-	-	-	-	-	
								6 weeks		-	-	-	-	-	-	
								8 weeks		-	-	-	-	-	No	
				-	No	-		2 weeks	No bacteria	-	-	-	-	No	-	
								4 weeks		-	-	-	-	-	-	
								6 weeks		-	-	-	-	-	-	
								8 weeks		-	-	-	-	-	Yes	
[70]	Zn–2Ag cylindric intramedullary nail	In vivo	Femoral condyles	MRSA	Yes	Soaked in implants	-	3 and 6 weeks	Very few bacteria in the surrounding bone tissue; no bacteria on the nail surface	Almost completely suppressed	Milder signs	-	-	-	-	Zn–2Ag alloy prevented MRSA infection and no osteomyelitis formation was observed, while promoting osseointegration.
	c.p. Ti cylindric intramedullary nail								Large number of bacteria on the nail surface and surrounding bone tissue	Yes	Yes	-	-	-	-	
	Zn–2Ag screw		Femoral condylar split-fracture	-	No	-	-	3 months	-	-	-	Yes	-	Yes	Yes	
	Ti–6Al–4V screw								-	-	-		-	No	No	
[77]	Mg-1Zn-0.5Sn rod	In vivo	Femoral condyle	*S. aureus* (ATCC 25923) [#] *E.coli* O157 (H7 NTCC 12900) [#]	No	No, Yes [#]	Penicillin postop, No [#]	1,7 and 14 days	Lowest number of bacterial colonies/smaller colonies [#]	-	-	-	Slower degradation rate *** [#]	-	-	Mg-1Zn-0.5Sn materials exhibited significant antibacterial ability compared to Ti materials. Mg-1Zn-0.5Sn had its degradation rate significantly reduced.
	Mg-1Zn rod								Low number of bacterial colonies/smaller colonies [#]	-	-	-	-	-	-	
	Ti rod [#]								Higher number of bacterial colonies [#]	-	-	-	-	-	-	

Notes: * These results report to visual examination after implant removal. ** After removal, the implant underwent SEM observation. *** Results obtained after emersion test. [#] Experiment results in vitro. [##] Chlorhexidine was used as an antiseptic solution to wash out the bone cavity before the implantation of bone substitutes.

## Data Availability

Not applicable.

## Supplementary Materials

The following supporting information can be downloaded at: https://www.mdpi.com/article/10.3390/bioengineering9080409/s1, Table S1. PRISMA checklist of the systematic search of the relevant studies; Table S2—Risk of bias analysis.

## Abbreviations

**(V-) BHA**—(Vancomycin-loaded) Bone-like hydroxyapatite/poly(amino acid); **Ag/n-HA/PU**—Nano-hydroxyapatite combined with a polyurethane containing silver; **AgNPs**—Silver nanoparticles; **AHRG (AgNPs-nHA@RGO)**—AgNP-loaded nHA@RGO 3D scaffolds; **Al**— Aluminum; **AMPs**—Antimicrobial peptides; **ANDB**—Antibiotic-eluting Nanofiber-loaded Deproteinized Bone; **AZ91**—Mg–9%Al–1%Zn; **β-TCP**—β-Tricalcium Phosphate; **BCP**—Bifasic-Calcium Phosfate; **Bio-Oss®**—Demineralized autologous graft bone; **BS**—Bare composite Scaffolds; **BV/TV**—Bone volume fraction (is the volume of mineralised bone per unit volume of the sample); **CaP**—Calcium Phosphate; **CFU**—Colony-Forming Units; **CMC**—Carboxymethyl Cellulose; **CoNS**—Coagulase-negative Staphylococci; **cpTi**—commercially pure Titanium; **CPX**—Ciprofloxacin; **Cu**—Copper; **DBBP**—Decellularized Bovine Bone Particles; **DPB**—Deproteinized Bovine cancellous Bone; **EPS**—Extracellular Polymeric Substance; **Ga**—Gallium; **HA**—Hydroxyapatite; **IAI**— Implant-associated infection; **IBDs**—Infected Bone Defects; **IMs**—Implantable Matrices; **JDBM**—3D-printed Mg–Nd–Zn–Zr implant; **K20(/100)**—Degradable Polyurethane, Hydroxyapatite and Decellularized Bovine Bone particles (loaded with vancomycin 100mg/mL); **Lev**—Levofloxacin; **LPC**—Large pore size 250-350μm; **Mg**—Magnesium; **Micro-CT**—Micro-Computed Tomography; **MM**—Medial Malleolar; **MRI**—Magnetic Resonance Imaging; **MRSA**—Methicillin-Resistant *Staphylococcus aureus;* **MSNs**—Mesoporous Silica Nanoparticles; **NaCl**—sodium chloride–a pore forming agent (porogen); **NanoHA OR n-HA**—Nanohydroxyapatite; **Nd**—Neodymium; **NDB**—Nanofiber-loaded Deproteinized Bone; **NPC**—Non-porous coat; **P3HB**—Poly-3-hydroxybutyrate; **PAA**—Poly(amino acid) - polymer of six amino acids, containing 6-aminocaproic acid, glycine, L-alanine, L-phenylalanine, L-proline and L-lysine; **PBS**—Phosphate-Buffered Saline; **PCL**—Polycaprolactone; **PEG 400**—Poly(ethylene glycol); **PLDLA**—Poly-L-D-Lactic Acid; **PLGA**—Poly (DL-lactic acid-co-glycolic acid); **PLLA**—Poly-L-lactic Acid; **PSI10**—Antimicrobial peptide RRWPWWPWRR-NH2; **PU**—Polyurethane; **RGO**—Reduced Graphene Oxide; **SA-V5(15)**—Scaffold absorbed with 5(15) wt% vancomycin (after the development of the scaffold); **SBF**—Simulated body fluid; **Se**—Selenium; **SE-V5(15)**—Scaffold entrapped with 5(15) wt% vancomycin (during scaffold synthesis); **Sn**—Tin; **SPC**—Porous coat of small pore size 150–250 μm; **Sr**—Strontium; **Tb.N**—Trabecular number; **Tb.Sp**—Trabecular spacing; **UMAO**—Ultrasonic Micro-Arc Oxidation; **(V-) BHA**—(Vancomycin-loaded) Bone-like hydroxyapatite/poly(amino acid); **Zn**—Zinc; **Zn-2Cu**—Alloy of Zinc and Cu mass ratios of 2wt%; **Zr**—Zirconium.
