# Supplementary material for: Biodegradable Bone Implants as a New Hope to Reduce Device-Associated Infections—A Systematic Review
Source: Bioengineering (Basel). 2022 Aug 22;9(8):409. doi: 10.3390/bioengineering9080409 (PMC9405200; doi:10.3390/bioengineering9080409)
Supplement: Supplementary file 1 [file bioengineering-09-00409-s001.zip › bioengineering-1829237-supplementary.pdf]

bioengineering

Supplementary Materials

Biodegradable Bone Implants as a New Hope to Reduce Device-Associated Infections—A Systematic Review

José C. C. Paiva [1], Luís Oliveira [2], Maria Fátima Vaz [3,4] and Sofia Costa-de-Oliveira [5,6,*]

1 Faculty of Medicine, University of Porto, 4200-319 Porto, Portugal
2 DPS—Product Systems Development, INEGI—Institute of Science and Innovation in Mechanical and Industrial Engineering, 4200-465 Porto, Portugal
3 IDMEC—Instituto Superior Técnico, Universidade de Lisboa, 1499-002 Lisboa, Portugal
4 Departamento de Engenharia Mecânica, Instituto Superior Técnico, Universidade de Lisboa, 1499-002 Lisboa, Portugal
5 Division of Microbiology, Department of Pathology, Faculty of Medicine, University of Porto, 4200-319 Porto, Portugal
6 Center for Health Technology and Services Research—CINTESIS@RISE, Faculty of Medicine, University of Porto, 4200-319 Porto, Portugal
* Correspondence: sqco@med.up.pt

Table S1. PRISMA 2020 checklist of the systematic search of the relevant studies.

Section/topic	#	Checklist item	Reported on page and paragraph/ table #
**TITLE**			
Title	1	Identify the report as a systematic review, meta-analysis, or both.	Page 1.
**ABSTRACT**			
Structured summary	2	Provide a structured summary including, as applicable: background; objectives; data sources; study eligibility criteria, participants, and interventions; study appraisal and synthesis methods; results; limitations; conclusions and implications of key findings; systematic review registration number.	Page 1.
**INTRODUCTION**			
Rationale	3	Describe the rationale for the review in the context of what is already known.	Pages 4-5, 8th and 9th paragraphs.
Objectives	4	Provide an explicit statement of questions being addressed with reference to participants, interventions, comparisons, outcomes, and study design (PICOS).	Page 5, 10th paragraph.
**METHODS**			
Protocol and registration	5	Indicate if a review protocol exists, if and where it can be accessed (e.g., Web address), and, if available, provide registration information including registration number.	-
Eligibility criteria	6	Specify study characteristics (e.g., PICOS, length of follow-up) and report characteristics (e.g., years considered, language, publication status) used as criteria for eligibility, giving rationale.	Page 6, 13th and 14th paragraphs.
Information sources	7	Describe all information sources (e.g., databases with dates of coverage, contact with study authors to identify additional studies) in the search and date last searched.	Page 5, 12th paragraph.

Search	8	Present full electronic search strategy for at least one database, including any limits used, such that it could be repeated.	Page 5, 12th paragraph.
Study selection	9	State the process for selecting studies (i.e., screening, eligibility, included in systematic review, and, if applicable, included in the meta-analysis).	Pages 6-7, 15th paragraph.
Data collection process	10	Describe method of data extraction from reports (e.g., piloted forms, independently, in duplicate) and any processes for obtaining and confirming data from investigators.	Pages 6-7, 15th paragraph.
Data items	11	List and define all variables for which data were sought (e.g., PICOS, funding sources) and any assumptions and simplifications made.	Pages 6-7, 15th paragraph. Page 8, Figure 2.
Risk of bias in individual studies / Risk of bias across studies	12/ 15	Describe methods used for assessing risk of bias of individual studies (including specification of whether this was done at the study or outcome level), and how this information is to be used in any data synthesis.	Page 7, 16th paragraph .
Summary measures	13	State the principal summary measures (e.g., risk ratio, difference in means).	Not applicable as this review does not include a meta-analysis.
Synthesis of results	14	Describe the methods of handling data and combining results of studies, if done, including measures of consistency (e.g., I^2) for each meta-analysis.	Not applicable as this review does not include a meta-analysis.
Additional analyses	16	Describe methods of additional analyses (e.g., sensitivity or subgroup analyses, meta-regression), if done, indicating which were pre-specified.	Not applicable as this review does not include a meta-analysis.
**RESULTS**			
Study selection	17	Give numbers of studies screened, assessed for eligibility, and included in the review, with reasons for exclusions at each stage, ideally with a flow diagram.	Pages 6-7, 15th paragraph. Page 7, Figure 1.
Study characteristics	18	For each study, present characteristics for which data were extracted (e.g., study size, PICOS, follow-up period) and provide the citations.	Page 10, Table 1a. Page 11, Table 1b. Pages 13-19, Table 2. Pages 26-29, Table 3.

Risk of bias within and across studies	19/ 22	Present data on risk of bias of each study and, if available, any outcome level assessment (see item 12).	Page 55, S2.
Results of individual studies	20	For all outcomes considered (benefits or harms), present, for each study: (a) simple summary data for each intervention group (b) effect estimates and confidence intervals, ideally with a forest plot.	Not applicable as this review does not include a meta-analysis.
Synthesis of results	21	Present results of each meta-analysis done, including confidence intervals and measures of consistency.	Not applicable as this review does not include a meta-analysis.
Additional analysis	23	Give results of additional analyses, if done (e.g., sensitivity or subgroup analyses, meta-regression [see Item 16]).	Not applicable as this review does not include a meta-analysis.
**DISCUSSION**			
Summary of evidence	24	Summarize the main findings including the strength of evidence for each main outcome; consider their relevance to key groups (e.g., healthcare providers, users, and policy makers).	Pages 33-40.
Limitations	25	Discuss limitations at study and outcome level (e.g., risk of bias), and at review-level (e.g., incomplete retrieval of identified research, reporting bias).	Pages 40-41, 70th paragraph.
Conclusions	26	Provide a general interpretation of the results in the context of other evidence, and implications for future research.	Page 41, 71st and 72nd paragraphs.
**FUNDING**			
Funding	27	Describe sources of funding for the systematic review and other support (e.g., supply of data); role of funders for the systematic review.	Page 42.

Table S2. Risk of bias analysis.

Ref.	Study design	1. Was administered dose or exposure level adequately randomized?	2. Was allocation to study groups adequately concealed?	3. Did selection of study participants result in appropriate comparison groups?	4. Did the study design or analysis account for important confounding and modifying variables?	5. Were experimental conditions identical across study groups?	6. Were research personnel blinded to the study group during the study?	7. Were outcome data complete without attrition or exclusion from analysis?	8. Can we be confident in the exposure characterization?	9. Can we be confident in the outcome assessment?	10. Were all measured outcomes reported?	11. Were there no other potential threats to internal validity?
80	*in vivo*	+	+	na	na	++	+	++	++	+	++	+
88	*in vivo*	+	+	na	na	++	+	++	+	+	++	+
81	*in vivo*	+	+	na	na	++	++	++	++	++	++	++
70	Case series	na	na	na	NR	na	na	na	NR	+	+	na
85	*in vivo*	NR	+	na	na	++	+	++	+	+	++	+
84	*in vivo*	NR	+	na	na	++	+	++	+	+	++	+
86	*in vivo*	NR	+	na	na	+	NR	++	+	+	++	+
90	*in vivo*	+	+	na	na	++	+	++	++	+	++	+
67	*in vivo*	+	+	na	na	++	+	++	++	+	++	+
79	*in vivo*	NR	NR	na	na	++	+	++	++	+	++	+
83	*in vivo*	+	+	na	na	+	+	++	+	+	+	+
75	*in vivo*	+	+	na	na	+	+	++	+	++	+	+
76	*in vivo*	+	+	na	na	++	+	++	++	+	++	++
78	*in vivo*	+	++	na	na	++	+	+	+	+	++	+
72	*in vivo*	NR	++	na	na	++	++	++	++	++	++	+
73	*in vivo*	+	++	na	na	+	+	+	+	+	+	+
71	Retrospective cohort study	na	na	++	+	na	na	++	+	++	++	++
74	*in vivo*	+	++	na	na	++	+	NR	+	+	++	+
87	*in vivo*	NR	++	na	na	++	+	+	+	+	++	+
68	*in vivo*	++	++	na	na	++	++	++	++	++	++	+
77	*in vivo*	+	++	na	na	+	+	+	++	+	++	+
69	*in vivo*	NR	++	na	na	+	+	+	++	+	++	NR

++	**Definitely Low risk of bias**: There is direct evidence of low risk-of-bias practices
+	**Probably Low risk of bias:** There is indirect evidence of low risk-of-bias practices OR it is deemed that deviations from low risk-of-bias practices for these criteria during the study would not appreciably bias results, including consideration of direction and magnitude of bias.
**NR**	**Probably High risk of bias:** There is indirect evidence of high risk-of-bias practices OR there is insufficient information (e.g., not reported or "NR") provided about relevant risk-of-bias practices
**-**	**Definitely High risk of bias:** There is direct evidence of high risk-of-bias practices
